# Supplementary figures and images for: Early Postnatally Induced Conditional Reelin Deficiency Causes Malformations of Hippocampal Neurons
Source: Biomolecules. 2025 Nov 28;15(12):1662. doi: 10.3390/biom15121662 (PMC12730948; doi:10.3390/biom15121662)

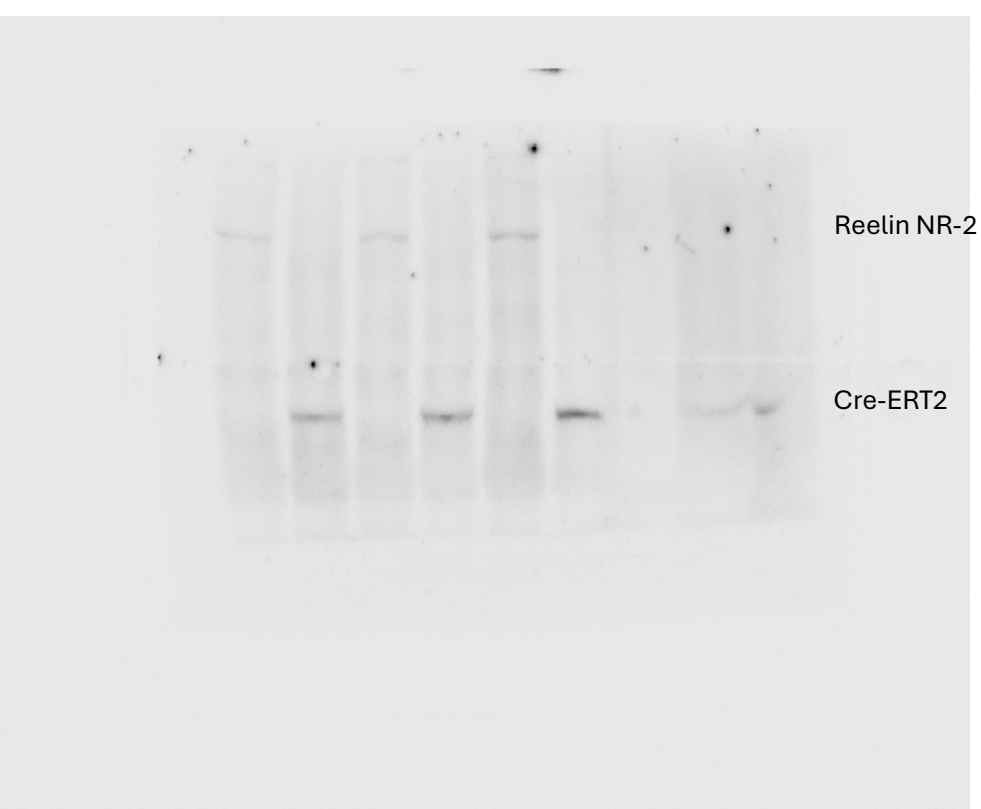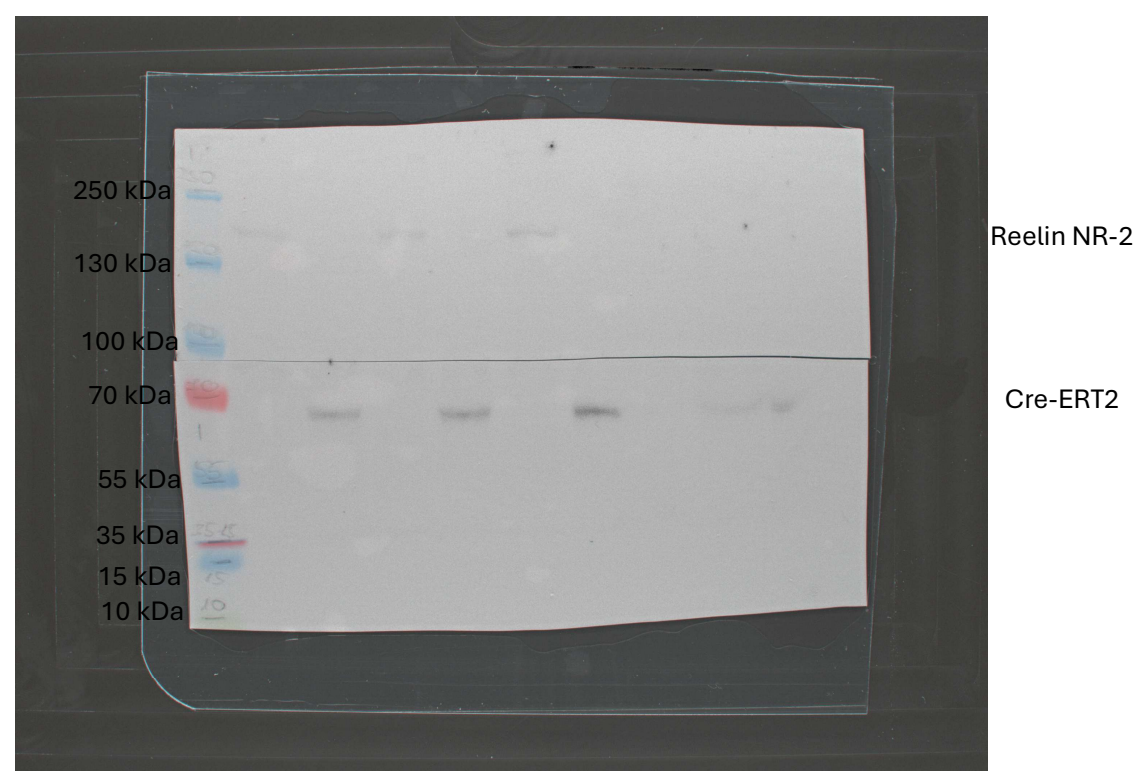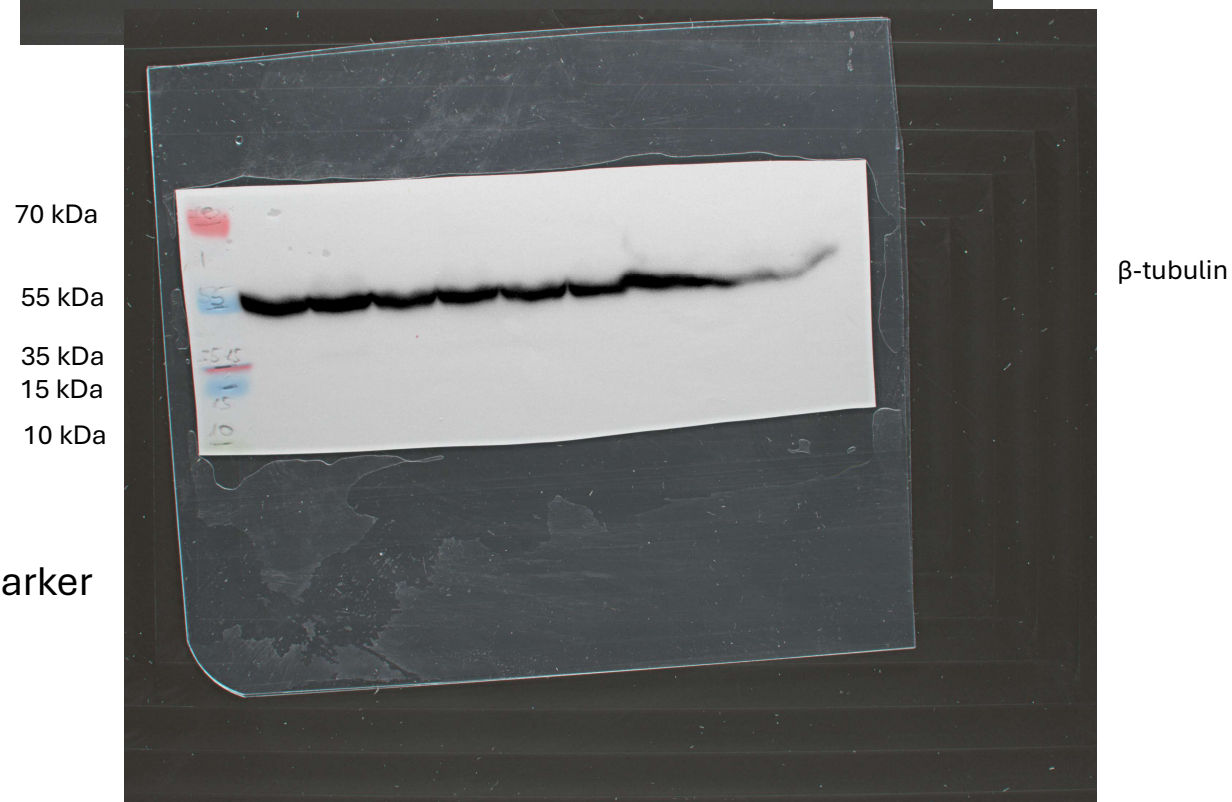

PageRuler Marker

Supplement: Supplementary file 1 [file biomolecules-15-01662-s001.zip › Original data reelin Western Blot 2 weeks of Figure 1D.pdf]

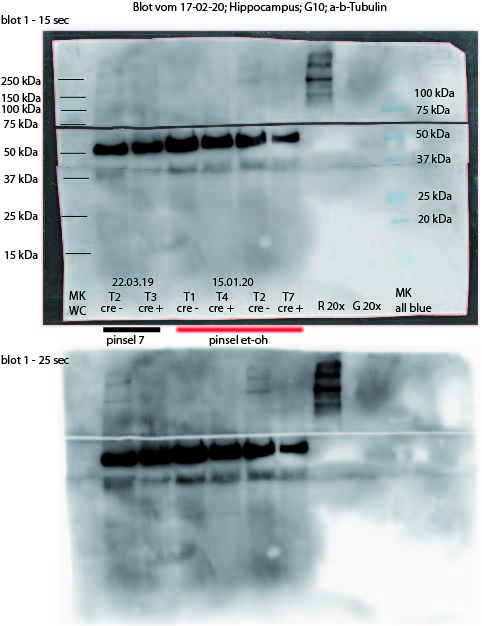

Supplement: Supplementary file 1 [file biomolecules-15-01662-s001.zip › Original data Western Blot 4 weeks of Figure 1E.jpg]
